# Reference Genes for Expression Analysis Using RT-qPCR in *Cnaphalocrocis medinalis* (Lepidoptera: Pyralidae)

**DOI:** 10.3390/insects13111046

**Published:** 2022-11-13

**Authors:** Xiaoyu Zhao, Jiawen Guo, Yanhui Lu, Tianyi Sun, Junce Tian, Jianlei Huang, Hongxing Xu, Zhengliang Wang, Zhongxian Lu

**Affiliations:** 1 College of Life Sciences, China Jiliang University, Hangzhou 310018, China; 2 State Key Laboratory for Managing Biotic and Chemical Threats to the Quality and Safety of Agro-Products, Institute of Plant Protection and Microbiology, Zhejiang Academy of Agricultural Sciences, Hangzhou 310021, China; 3 College of Plant Protection, Nanjing Agricultural University, Nanjing 210095, China; 4 College of Agriculture and Forestry, Hebei North University, Zhangjiakou 075000, China

**Keywords:** reference genes, migratory insect, *Cnaphalocrocis medinalis*, RT-qPCR, expression stability

## Abstract

**Simple Summary:**

The reference gene is the key to verifying the relative expression of target genes. However, the expression of common housekeeping genes is not stable under different experimental conditions, which may lead to misleading gene expression results. In this study, the stability of thirteen housekeeping genes of the rice pest *Cnaphalocrocis medinalis* at different developmental stages, larvae tissues, rice feedings, temperature treatments, and adult ages, nutritional conditions, mating statuses and different take-off characteristics was identified. Finally, the relative expression of *Trypsin-3* in different rice varieties was evaluated to verify the reliability of the results. Our results will help to improve the accuracy of RT-qPCR analysis and lay a foundation for the analysis of target gene expression for *C. medinalis* in the future.

**Abstract:**

*Cnaphalocrocis medinalis* is a destructive migratory rice pest. Although many studies have investigated its behavioral and physiological responses to environmental changes and migration-inducing factors, little is known about its molecular mechanisms. This study was conducted to select suitable RT-qPCR reference genes to facilitate future gene expression studies. Here, thirteen candidate housekeeping genes (*EF1α*, *AK*, *EF1β*, *GAPDH*, *PGK*, *RPL13*, *RPL18*, *RPS3*, *18S rRNA*, *TBP1*, *TBP2*, *ACT*, and *UCCR*) were selected to evaluate their stabilities under different conditions using the ∆CT method; the geNorm, NormFinder, BestKeeper algorithms; and the online tool RefFinder. The results showed that the most stable reference genes were *EF1β*, *PGK*, and *RPL18*, related to developmental stages; *RPS3* and *RPL18* in larval tissues; *EF1β* and *PGK* in larvae feeding on different rice varieties; *EF1α*, *EF1β*, and *PGK* in larvae temperature treatments; *PGK* and *RPL13*, related to different adult ages; *PGK*, *EF1α*, and *ACT*, related to adult nutritional conditions; *RPL18* and *PGK*, related to adult mating status; and, *RPS3* and *PGK*, related to different adult take-off characteristics. Our results reveal reference genes that apply to various experimental conditions and will greatly improve the reliability of RT-qPCR analysis for the further study of gene function in this pest.

## 1. Introduction

Real-time quantitative polymerase chain reaction (RT-qPCR) is a method for analyzing specific gene expression that is widely used because of its high sensitivity, high accuracy, specificity, and rapid response [1,2]. When RT-qPCR relatively quantifies the change in the gene expression level, the stably expressed reference gene is most commonly used as the internal control for data normalization [3]. Therefore, finding the appropriate reference gene is an important step of RT-qPCR detection [4,5]. Some housekeeping genes are often used as reference genes of RT- qPCR, such as *β-actin (ACT)*, *Glyceraldehyde-3-Phosphate dehydrogenase (GAPDH)*, *18S ribosomal RNA* (*18S rRNA*), and *β-Tubulin (β-TUB)* [6,7,8,9,10]. However, studies on the expression of reference genes in different species and under different experimental conditions have shown that most housekeeping genes do not keep stability [4,6,9]. The unstable expression of the reference genes may lead to misleading gene expression results [11]. Moreover, a single reference gene cannot fully satisfy all experimental requirements [12]. Therefore, it is necessary to screen and validate the reference genes for different species under specific experimental conditions [13].

The rice leaf folder, *Cnaphalocrocis medinalis* (Lepidoptera: Pyralidae), is prominent in the Asian rice region [14]. Its strong adaptability to hosts and adverse stresses makes it one of the most destructive pests on rice [15]. Furthermore, the strong migratory ability of the *C. medinalis* moths has facilitated the expansion of their geographical distribution, with outbreaks in many rice-growing areas in Asia [16,17,18]. The population outbreaks are sudden and dependent on immigrating population characteristics, which makes their accurate prediction and control more difficult [19]. Therefore, the environmental adaptations and migratory mechanisms of this pest need to be studied in depth. Although many studies have been performed on behavioral responses to changes in abiotic conditions such as temperature and light and the factors including migration [20,21], little is known about the molecular mechanisms that regulate these behavioral and physiological changes in this species. Studies on gene expression and its regulation may help to further understand the environmental adaptations and migration mechanisms of the *C. medinalis*. It is important to screen the reference genes of *C. medinalis* suitable for different experimental conditions as the data normalization standard to analyze the relative expression of related genes. In the current study, *β-actin* was mostly chosen as the reference gene for *C. medinalis* [22,23,24,25,26]. However, the expression of *β-actin* has been found to be unstable in different tissues and developmental stages in other insects, such as *Chlorops oryzae* [27] and *Galeruca daurica* (Joannis) [28]. Therefore, it is important to determine the best reference gene for *C. medinalis* under specific conditions.

In this study, the expression stability of thirteen candidate reference genes was assessed in *C. medinalis*, including *Elongation factor 1 α (EF1α), Arginine kinase (AK), Elongation factor 1 β (EF1β), Glyceraldehyde-3-Phosphate dehydrogenase (GAPDH), Phosphoglycerate kinase (PGK), Ribosomal protein L 13 (RPL13), Ribosomal protein L 18 (RPL18), Ribosomal protein S 3 (RPS3), 18S ribosomal RNA (18S rRNA), TATA-box binding protein 1 (TBP1), TATA-box binding protein 2 (TBP2), β-actin (ACT)*, and *Ubiquinol-cytochrome c reductase (UCCR).* The expression levels of the genes at different development stages, larval tissues, larvae feeding on rice varieties, larvae temperature treatments, adult ages, adult nutritional conditions, adult mating statuses and adult take-off characteristics were analyzed using ΔCt, geNorm, NormFinder, and BestKeeper [29,30,31]. Then, a comprehensive ranking for each experimental condition was generated using RefFinder based on the rankings created by the four statistical algorithms [32]. Our results will provide valuable information for quantitatively detecting gene expression using RT-qPCR for further research on the molecular mechanisms of the environmental adaptation and migration of *C. medinalis*.

## 2. Materials and Methods

### 2.1. Rice Plant Preparing and Insect Rearing

Four rice varieties, namely TN1 (TN1, seeds provided by the China National Rice Research Institute), Yongyou 1540 (Ningbo Seed Co., Ltd., Ningbo, China), Xiushui 134 and Zhongzao 39 (Zhejiang Wuwangnong Seeds Shareholding Co., Ltd., Hangzhou, China), were grown in a greenhouse at the Zhejiang Academy of Agricultural Sciences in Hangzhou, China (30.31° N, 120.20° E), without any insect pests or pesticide treatments and were used for feeding insects 45 days after germination. All the insects were reared in RXZ intelligent artificial climate chambers (Ningbo Jiangnan Instrument Factory, Ningbo, Zhejiang, China) at 26 ± 1 °C, 80 ± 5% relative humidity, and a photoperiod of 14:10 L:D [33]. Unless otherwise stated, the temperature, humidity, and light conditions of the experiments below were the same as the rearing conditions.

*C. medinalis* larvae were collected from Nanjing, Jiangsu Province, in 2019 (118.78° E, 32.06° N) and were reared on wheat seedlings until pupation [34]. Pupae were removed and transferred into a plastic box (16 cm × 24 cm × 22 cm in length, width, and height, respectively), and the bottom of the box was filled with moist cotton to maintain high relative humidity. After emergence, 10 adults (female: male = 1:1) were transferred to a 500 mL plastic cup with absorbent cotton wool soaked in 5% honey solution as a supplemental nutrient for adults. The top of the cup was covered with plastic film for adults to oviposit on. TN1 rice was used for larval rearing, a *C. medinalis*-susceptible variety [35].

### 2.2. Experimental Treatments

#### 2.2.1. Developmental Stages

*C. medinalis* individuals at different development stages (first to fifth instar larvae, 4-day-old female/male pupae, and 2-day-old female/male adults) were randomly collected. The sample sizes were 50, 20, 10, 5, and 3 per replications for the 1st to 4th instar larvae and other developmental stages, respectively. Three times were replicated for each treatment. Samples were immediately frozen in liquid nitrogen and stored at −80 °C until use. Samples were collected and stored using this method where not specifically described below. Three biological replicates were set for all the following experimental treatments.

#### 2.2.2. Larval Tissues

Different larval tissues were collected by dissecting 5th instar larvae with reference to the method of Zhang et al. [11]. The larvae were first placed on ice, and the head and tail of the larvae were cut off with dissecting scissors. Afterward, the larval gut was pulled out, and the contents were gently scraped out with dissecting forceps. One end of the larval body was then held in place with forceps, and the fat body was scraped out with another forceps. The head, gut, fat body, and epidermis of the larvae were collected separately. A total of 90 larvae were dissected.

#### 2.2.3. Larvae Feeding on Different Rice Varieties

Four rice varieties, namely, TN1, Yongyou 1540 (Ningbo Seed Co., Ltd., Ningbo, China), Xiushui 134, and Zhongzao 39 (Zhejiang Wuwangnong Seeds Shareholding Co., Ltd., Hangzhou, China), were used for this experiment. Larvae were reared on these four rice varieties after hatching and were used for the experiment when they reached the third instar. Ten larvae were collected as biological replicates.

#### 2.2.4. Larvae Temperature Treatments

A total of seventy-five fourth instar larvae, which were subjected to temperatures of 16, 21, 26, 31, and 36 °C for 1 h in a GXZ intelligent light incubator (Ningbo Jiangnan Instrument Factory, Ningbo, Zhejiang, China), were collected. For each treatment, 5 individuals were used as one replicate.

#### 2.2.5. Adult Ages

Five pairs of newly emerged adults were placed in a 500 mL plastic cup with cotton soaked in a 5% honey solution placed in the bottom and the top of the cup sealed with plastic film. Since most studies focus more on the reproduction and migration of *C. medinalis*, and their peak periods of reproduction and migration are from the first to five days after emergency [36,37], male and female moths from 1 to 5 days of age were collected for this experiment. There were three replicates at each age and three moths per replicate.

#### 2.2.6. Adult Nutritional Conditions

*C. medinalis* adults on the day of emergence were divided into two treatment groups: (i) feeding group: one male moth and one female moth were fed with 5% honey solution from the first day after emergence; (ii) starvation group: one pair of moths were fed with water from the first day after emergence; no other food was provided [17]. Two-day-old moths after different nutrient treatments were collected in triplicate (3 moths per replicate).

#### 2.2.7. Adult Mating Statuses

*C. medinalis* adults were divided into two treatment groups after emergence: (i) mating group: a female moth and a male moth were paired for mating; and (ii) virgin group: female moths and male moths that were not mated were raised separately [17]. Samples (3 adults per replicate) were collected after 3 days.

#### 2.2.8. Different Adult Take-Off Characteristics

Two-day-old female adults were collected and placed in the take-off behavior observation device. The take-off behavior observation device adopted a cylindrical take-off cage made of highly transparent PVC film (a diameter of 50 cm and a height of 120 cm). The bottom of the take-off cage was a white plastic plate, and a 500 mL transparent plastic cup was placed in the middle as the take-off platform. A distinction was made between migratory and resident moths according to different adult take-off characteristics. Moths that took off at a vertical distance greater than 100 cm were considered migratory moths, and those that remained stationary or hovered at an altitude of less than 100 cm were considered resident moths [18]. For each type, 3 female individuals were used as one replicate.

### 2.3. Total RNA Isolation and cDNA Synthesis

Total RNA was isolated from each sample using TRIzol reagent (Tiosbio, Beijing, China), and the purity and concentration of RNA were determined on a NanoDrop2000 (Thermo Fisher Scientific, Waltham, MA, USA). The RNA samples with absorbance ratios of A_260_/A_280_ around 2.0 were selected for further analysis. The extracted RNA was digested by DNase I (TaKaRa, Beijing, China) to remove genomic DNA contamination [38]. Finally, 1 μg of total RNA was used to synthesize cDNA using a 1st cDNA Synthesis Kit (gDNA removal) (Tiosbio, Beijing, China). The cDNA was applied to both PCR and RT-qPCR.

### 2.4. Selection of Candidate Reference Genes and Primer Design

Thirteen candidate genes, namely *EF1α*, *AK*, *EF1β*, *GAPDH*, *PGK*, *RPL13*, *RPL18*, *RPS3*, *18S rRNA*, *TBP1*, *TBP2*, *UCCR*, and *ACT*, were selected from the literature. The primers of *EF1α*, *AK*, *EF1β*, *GAPDH*, *PGK*, *RPL13*, *RPL18*, *RPS3*, *18S rRNA*, *TBP1*, *TBP2*, and *UCCR* were designed based on the genome data of *C. medinalis* (http://v2.insect-genome.com/Organism/192, accessed on 6 June 2021). The Gene ID of these genes in the genome is Cmed07334, Cmed10701, Cmed08616, Cmed11239, Cmed22532, Cmed14502, Cmed03810, Cmed05991, Cmed07228, Cmed19702, Cmed03377, and Cmed15494 [39]. Except for genes from genome data, *ACT* (GenBank accession number: JN029806.1) was also added as a potential candidate reference gene. The design and quality evaluation of all primers were performed using Oligo 7, and primer sequences are listed in Table 1. 

### 2.5. RT-qPCR

RT-qPCR reactions were carried out on a CFX-96 real-time PCR system (BioRad, Hercules, CA, USA). Reactions were conducted in a 20 μL mixture containing 10 μL of 2 × Kappa SYBR Green I qPCR Mix (with ROX) (Tiosbio, Beijing, China), 1 μL of cDNA, 1 μL of each primer, and 7 μL of RNase-free water. The reaction conditions were as follows: initial denaturation at 95 °C for 30 s, followed by 40 cycles at 95 °C for 5 s, 60 °C for 34 s, and 72 °C for 15 s. A melting curve analysis was conducted in the 60–95 °C temperature range to ensure the specificity of the primers. Three technical replicates were set up for each biological replicate. A standard curve was generated from the five-fold dilution series of cDNA, the slopes were analyzed, and the corresponding amplification efficiencies were calculated by Formula (1) [40].

(1) $$E=\left(10^{-\frac{1}{slope}}-1\right)*100\%\;$$

### 2.6. Expression Stability of Candidate Reference Genes under Different Treatments

The stability of each candidate reference gene was calculated by the geNorm, NormFinder, BestKeeper, and ∆Ct methods and comprehensively ranked by RefFinder (http://blooge.cn/RefFinder/, accessed on 22 July 2022). The optimal number of reference genes used for normalizing the target gene was determined by the variation value (V_n_/V_n+1_) calculated by geNorm. V_n_/V_n+1_ ≤ 0.15 indicated that the number of optimal reference genes for normalization was n [41,42].

### 2.7. Verification of Reference Gene

The *Trypsin-3* (*Try3*) of *C. medinalis* was selected as the target gene to verify the stability of candidate reference genes. The primer sequence of the target gene was as follows: forward (5′-AACTTCAAGAAGCCGTCGAA-3′) and reverse (5′-ATGATAAACCCGCCACAGAA-3′). The average relative expressions of *Try3* in different rice feeding were computed based on the 2^−ΔΔCt^ method and from three replicates [43]. TN1 was selected as control because it was susceptible to *C. medinalis* infestation [35]. The gene expression levels under different treatments were analyzed by one-way ANOVA and compared using Tukey’s honestly significant difference test (Tukey’s HSD). All statistical analyses were performed using SPSS 20.0 software (IBM, Armonk, NY, USA).

## 3. Results

### 3.1. Total RNA Quality and Amplification Efficiencies

The A_260_/A_280_ ratios ranged from 1.80 to 2.11, showing that the RNA samples were of good quality. Agarose gel electrophoresis (Figure S1) showed that the amplified fragments of all the primers were 122–163 bp in length, and melting curve analysis (Figure S2) using the RT-qPCR of the thirteen candidate reference genes had a single peak, indicating the good specificity of the primers. The PCR efficiency (E) and the regression coefficient (R^2^) were calculated using the slope of the standard curve established for each primer pair. The E-values ranged from 102% (*ACT*) to 119% (*UCCR*), which was within the required range of 80.0–120.0% (Table 1). The regression coefficient ranged from 0.982 (*TBP1*) to 0.999 (*PGK* and *UCCR*) (Table 1). These results indicated that the selected quantitative primer pairs were well designed and had good amplification efficiency and specificity. All primers met the requirements of quantitative fluorescence analysis and were suitable for quantifying the candidate reference genes.

### 3.2. Expression Profiles of Candidate Reference Genes

**Figure 1:**
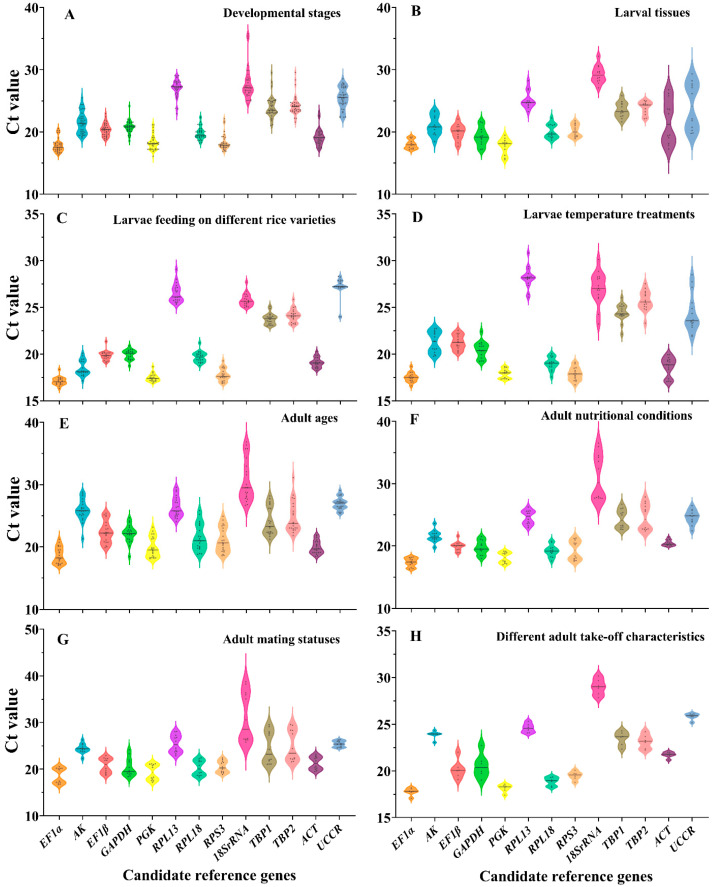
Expression profiles of candidate reference genes under eight experimental conditions. (**A**) developmental stages; (**B**) larval tissues; (**C**) larvae feeding on different rice varieties; (**D**) larvae temperature treatments; (**E**) adult ages; (**F**) adult nutritional conditions; (**G**) adult mating statuses; (**H**) different adult take-off characteristics. Lines across the Violin plots depict the medians of Ct values. Black dots represent measured values (jitter effect was applied to avoid overplotting).

The expression of the thirteen candidate reference genes under the different experimental conditions was evaluated according to the threshold cycle (Ct) values. The gene expression analysis of the thirteen candidate reference genes in all samples under eight conditions showed a range of Ct means of 15.54–38.75 (Figure 1), indicating significant differences in expression profiles (developmental stages: *F*_12, 390_ = 139.228, *p* < 0.001; larval tissues: *F*_12, 143_ = 37.683, *p* < 0.001; larvae feeding on different rice varieties: *F*_12, 247_ = 554.828, *p* < 0.001; larvae temperature treatments: *F*_12, 182_ = 171.332, *p* < 0.001; adult ages: *F*_12, 260_ = 65.584, *p* < 0.001; adult nutritional conditions: *F*_12, 143_ = 73.934, *p* < 0.001; adult mating statuses: *F*_12, 143_ = 25.351, *p* < 0.001; different adult take-off characteristics: *F*_12, 65_ = 143.929, *p* < 0.001). At developmental stages, *EF1α* and *RPS3* had smaller gene expression variations. Across larvae feeding on different rice varieties and larval tissues, *EF1α* and *PGK* had the smallest gene expression variation. Among larvae temperature treatments, the fluctuation of *EF1α* expression was the smallest (Figure 1A–D). In the four treatments of adults, the expression of *18S rRNA* fluctuated significantly (Figure 1E–G). Overall, *EF1α* was the most abundant gene, and *18S rRNA* was the least expressed gene.

### 3.3. Stability of Candidate Reference Genes in C. medinalis under Different Experimental Conditions

#### 3.3.1. Developmental Stages

**Figure 2:**
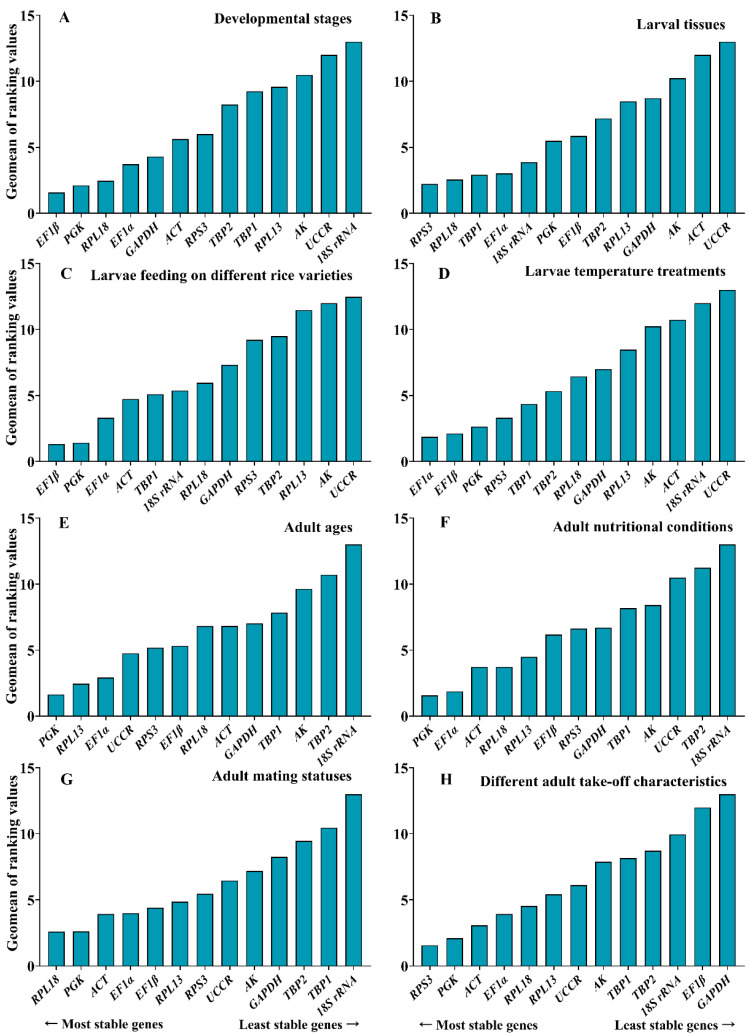
Expression stability of the candidate reference genes under eight experimental conditions calculated by RefFinder: (**A**) developmental stages; (**B**) larval tissues; (**C**) larvae feeding on different rice varieties; (**D**) larvae temperature treatments; (**E**) adult ages; (**F**) adult nutritional conditions; (**G**) adult mating statuses; (**H**) different adult take-off characteristics. A lower Geomean order indicated more stable expression.

**Figure 3:**
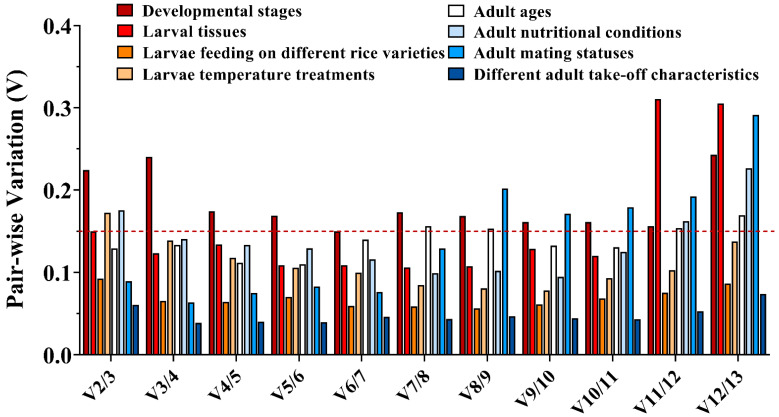
Determination of optimal number of normalization factors in *C. medinalis* under eight experimental treatments. The pairwise variation (V_n_/V_n+1_) was analyzed by geNorm algorithm. When the V value is below 0.15, there is no need to add additional reference genes for normalization.

The least stable gene evaluated by four algorithms was *18S rRNA*. *EF1β* was the most stable gene in ∆Ct and geNorm, *GAPDH* was the most stable in BestKeeper, and *RPL18* was the most stable in NormFinder (Table 2). The stability of the thirteen reference genes was ranked by RefFinder, from high to low: *EF1β* > *PGK* > *RPL18* > *EF1α* > *GAPDH* > *ACT* > *RPS3* > *TBP2* > *TBP1* > *RPL13* > *AK* > *UCCR* > *18S rRNA* (Figure 2). Pair-wise variation analysis of reference genes showed that V_6/7_ was less than 0.15 (Figure 3), indicating that gene expression analysis required six different reference genes in the developmental stage. Based on the above comprehensive ranking, we recommended the following six genes as reference genes in developmental stages: *EF1β*, *PGK, RPL18*, *EF1α*, *GAPDH,* and *ACT*.

#### 3.3.2. Larval Tissues

For different larval tissues, the evaluation of the most stable gene was different: *RPL18* was the most stable gene in ∆Ct, *EF1α* was the most stable gene in BestKeeper, *RPS3* was the most stable gene in NormFinder, and *18S rRNA* and *TBP1* were the most stable genes in geNorm, but the least stable gene in the four algorithms was *UCCR* (Table 2). Combining the four algorithms, the comprehensive ranking by RefFinder was as follows: *RPS3* > *RPL18* > *TBP1* > *EF1α* > *18S rRNA* > *PGK* > *EF1β* > *TBP2* > *RPL13* > *GAPDH* > *AK* > *ACT* > *UCCR* (Figure 2). V_2/3_ was around 0.15 in geNorm (Figure 3); this suggested that two genes should be selected as reference genes in subsequent studies on other genes in larval tissues. Here, we recommended *RPS3* and *RPL18* as reference genes.

#### 3.3.3. Larvae Feeding on Different Rice Varieties

All analyses except for Bestkeeper indicated that *EF1β* was the most stable gene, while BestKeeper considered *PGK* as the most stable gene (Table 2). The stability of the RefFinder comprehensive evaluation was from high to low: *EF1β* > *PGK* > *EF1α* > *ACT* > *TBP1* > *18S rRNA* > *RPL18* > *GAPDH* > *RPS3* > *TBP2* > *RPL13* > *AK* > *UCCR* (Figure 2). The analysis of pair-wise variation showed that V_2/3_ was less than 0.15 (Figure 3), and the calculation of two genes as reference genes (*EF1β* and *PGK*) was accurate enough.

#### 3.3.4. Larvae Temperature Treatments

Under temperature–stress conditions, ∆Ct and NormFinder suggested that *EF1β* was the most stable gene, and BestKeeper and geNorm indicated that *EF1α* was one of the most stable genes in larvae, whereas the least stable gene was *UCCR* (Table 2). The stability order of the thirteen reference genes was ranked as follows: *EF1α* > *EF1β* > *PGK* > *RPS3* > *TBP1* > *TBP2* > *RPL18* > *GAPDH* > *RPL13* > *AK* > *ACT* > *18S rRNA* > *UCCR* (Figure 2). The variation in V_3/4_ was less than 0.15 (Figure 3), indicating that gene expression analysis required three different reference genes under different temperature treatments: *EF1α*, *EF1β*, and *PGK*.

#### 3.3.5. Adult Ages

Besides BestKeeper suggesting that *UCCR* was the most stable gene, the other three algorithms revealed that *PGK* was the most stable gene at different adult ages (Table 2). The RefFinder evaluation found that *PGK* ranked the highest in terms of stability, followed by *RPL13*, and *18S rRNA* was the lowest in terms of stability (Figure 2). The variation value V_2/3_ was less than 0.15 (Figure 3). Therefore, it was recommended to use two reference genes (*PGK* and *RPL13*) to detect the expression level of target genes at different adult ages.

#### 3.3.6. Adult Nutritional Conditions

Based on the results of the three algorithms (∆Ct, NormFinder, and geNorm), *PGK* was identified as the most stable gene in nutritional status, but the BestKeeper analysis showed that *ACT* had the highest expression stability (Table 2). RefFinder ranked the selected housekeeping genes in the following order from the most to the least stable: *PGK > EF1α > ACT > RPL18 > RPL13 > EF1β > RPS3 > GAPDH > TBP1 > AK > UCCR > TBP2 > 18S rRNA* (Figure 2). In addition, the pair-wise variance value V_3/4_ was less than 0.15 in geNorm analysis (Figure 3). We thus suggest that, under the same experimental conditions, using three different reference genes (*PGK*, *EF1α*, and *ACT*) to calculate the relative expression of target genes is more accurate.

#### 3.3.7. Adult Mating Statuses

*RPL18* was the most stable gene evaluated by ∆Ct and NormFinder, and *UCCR* was the most stable gene in BestKeeper, *EF1α,* and *PGK* in geNorm in adults at different mating statuses. Additionally, all algorithms suggested that *18S rRNA* was the least stable gene (Table 2). High stability to low stability in RefFinder is ranked as follows: *RPL18 > PGK > ACT > EF1α > EF1β > RPL13 > RPS3 > UCCR > AK > GAPDH > TBP2 > TBP1 > 18S rRNA* (Figure 2). Comparing two pairs of variation values found that V_2/3_ was less than 0.15 (Figure 3). Therefore, two different genes should be used as reference genes. Combined with the order of RefFinder, *RPL18* and *PGK* were the best choices to detect the expression level of the target gene in adults with different mating statuses.

#### 3.3.8. Different Adult Take-Off Characteristics

The evaluation results of ∆Ct, NormFinder, and geNorm showed that *RPS3* was one of the most stable genes, while Best Keeper considered *ACT* as the most stable gene (Table 2). According to the results of RefFinder, the stability was ranked as *RPS3 > PGK > ACT > EF1α > RPL18 > RPL13 > UCCR > AK > TBP1 > TBP2 > 18S rRNA >EF1β > GAPDH* (Figure 2). The variation in V_2/3_ was less than 0.15 (Figure 3). This showed that, under the same experimental conditions, at least two different genes were required as reference genes to verify the relative expression of target genes. Based on the ordering of RefFinder, we considered *RPS3* and *PGK* as the most appropriate reference gene combinations.

### 3.4. Validation of Reference Genes with Try3

**Figure 4:**
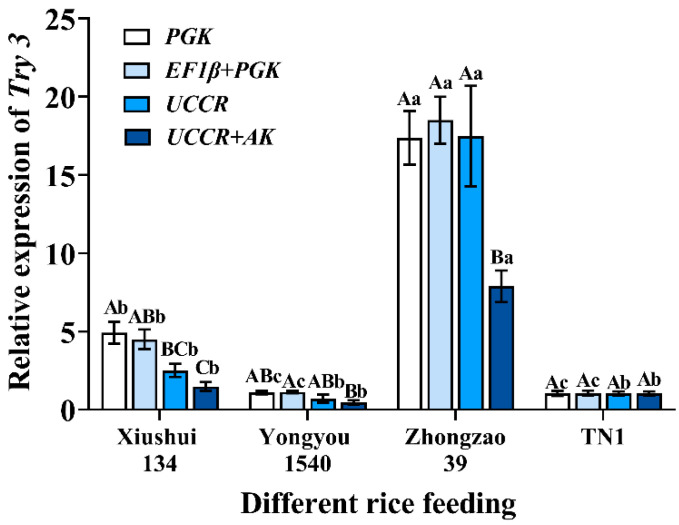
Expression of *Try3* gene under different rice feeding using validated reference genes for data normalization. Four reference gene combinations (*PGK*, *EF1β* + *PGK*, *UCCR*, *UCCR* + *AK*) were used for the normalization. The data in the figure were the mean ± standard error. Different lowercase letters indicate that, after normalization with the same reference gene, there was a significant difference in the expression level of *Try3* in larvae of *C. medinalis* feeding on different rice varieties (Tukey’s HSD-*p* < 0.05). Different uppercase letters indicate that there were significant differences in the normalization results of each reference gene (Tukey’s HSD-*p* < 0.05).

To evaluate the stability of the selected reference genes, we analyzed the expression level of *Try3* in the third instar *C. medinalis* larvae fed on different rice varieties. The following reference genes were used to normalize: *PGK, PGK + EF1β* (the most stable reference gene), and *UCCR*, *UCCR* + *AK* (the least stable reference gene). The highest accumulated level of *Try3* was found in larvae fed on Zhongzao 39. The expression of *Try3* in larvae fed by Xiushui 134 was significantly up-regulated as analyzed by *PGK* and *PGK + EF1β*. However, there was no significant difference among the larvae feeding on Xiushui 134, Yongyou 1540, and TN1 after analysis with *UCCR* and *UCCR + AK* (*PGK*: *F*_3, 16_ = 69.372, *p* < 0.001; *PGK + EF1β*: *F*_3, 16_ = 103.448, *p* < 0.001; *UCCR*: *F*_3, 16_ = 24.709, *p* < 0.001; *UCCR + AK*: *F*_3, 16_ = 42.219, *p* < 0.001). Except for TN1, the *Try3* expression levels of the larvae fed by the other three varieties of rice showed significant differences with different reference gene combinations (Xiushui 134: *F*_3, 16_ = 9.298, *p* = 0.001; Yongyou 1540: *F*_3, 16_ = 4.090, *p* = 0.025; Zhongzao 39: *F*_3, 16_ = 6.018, *p* = 0.006; TN1: *F*_3, 16_ = 0.004, *p* = 1.000) (Figure 4). This shows that the stability and reliability of the results are reduced when using unstable reference genes or combinations.

## 4. Discussion

RT-qPCR is the most widely used gene expression detection method, but its accuracy and reliability depend on the normalization of data by stable reference genes [44]. To avoid data fuzziness, each candidate housekeeping gene needs to be verified under certain experimental conditions [28]. In our study, the expression stability of thirteen candidate reference genes in *C. medinalis* was assessed at different developmental stages, larvae tissues, larvae feeding on rice varieties, larvae temperature treatments, adult ages, take-off characteristics, mating statuses, and nutritional conditions. Our data showed that there was no single reference gene suitable for all the conditions. The results obtained by screening appropriate reference genes for specific conditions were more reliable than using common housekeeping genes directly.

The comprehensive orders with the online tool RefFinder showed significant differences among different experimental conditions. This phenomenon was also found in other insects, such as *Miscanthus sacchariflorus* [1], *Apis mellifera* [3], and *Luffa cylindrica* [4]. Our results showed *EF1β* was the most suitable reference gene under different varieties of rice feeding, which was the same as *Chrysomya megacephala* [45]. Additionally, in larval tissues, *RPS3* was a stable reference gene. This result was confirmed in *Sesamia inferens* [46], *Ips typographus* [47], and *Tribolium castaneum* [48], suggesting that *RPS3* may be used as the reference gene for most insects in different tissues. Nevertheless, not all reference genes applicable to one insect could be used as reference genes for other insects. For example, *18S rRNA* and *GAPDH* are often used as reference genes in many insects [13]. However, in our experiments, *18S rRNA* was the least stable gene at different developmental stages and in adults in the other three conditions, except for different adult take-off characteristics, which may be due to the fact that the *C. medinalis* moth is a migratory insect and its cellular rRNA levels may be more susceptible to external environments, such as nutrient deficiencies [49]. In addition, we found that *GAPDH* had the lowest stability with different take-off characteristics, probably because the process of migration or take-off requires energy [50], and *GAPDH* is closely related to energy metabolism [51]. These results indicated that “classic” genes were variable and needed to be assessed before further use as reference genes. Therefore, it is necessary to screen and verify the reference genes of *C. medinalis* under other different conditions more comprehensively.

Previous studies have found that some genes could be used as universal reference genes under multiple conditions. *ACT* was a stable gene in *Diaphania caesalis* [41], and *Aphis gossypii* [52] and *α-Tublin* could be used as a reference gene in *Empoasca onukii* Matsuda [53] and *Anthonomus eugenii* Cano [54] under diverse conditions. We found that the expression of *EF1α* and *EF1β* was relatively stable in the other three conditions of *C. medinalis* larvae, except for feeding on different rice varieties, which means that they were used as references for the larval stage of *C. medinalis.* The stability of the two genes was also confirmed under diverse conditions in *Cydia pomonella* [5] and *Phthorimaea operculella* [9] larvae. They may be used as reference genes for studying Lepidoptera larval-stage-related physiology. More interestingly, *PGK* showed relatively stable expression under seven experimental conditions, except for larval tissues (comprehensively ranking in the top three under these seven conditions). *PGK* is the key enzyme of glycolysis, which plays a major role in organism survival, and its sequence is highly conservative [55]. The amount of *PGK* mRNA expression is high, and the mRNA content in the larval and adult stages follows the classical transcription pattern of enzymes related to general metabolic pathways [56]. Furthermore, *PGK* was one of the reference genes in *Aedes albopictus* early embryos [57]. Therefore, we believe that *PGK* can be used as a reference gene to determine the expression of the target gene in *C. medinalis* under most physiological conditions.

To validate our findings, we analyzed the expression of *Try3* in response to different varieties of rice feeding. *Try3* is an important enzyme for digesting protein in insect guts [58]. After normalization with *EF1β + PGK* and *UCCR*, the results of *Try3* expression were different. This result suggests that it is important to select appropriate reference genes to standardize the expression of target genes. Notably, two or more reference genes are often used for more accurate quantitative analysis [59]. The number of reference genes used to verify the expression of the target gene was one, two, three, or more in insects [60,61,62]. Many studies suggested that more than one stably expressed reference gene should be used, as the selection of multiple reference genes helps to reduce the deviation of data normalization [63]. Our study also found that the number of recommended reference genes under different experimental conditions ranged from two to six. Nevertheless, in past studies, most research on *C. medinalis* used a single reference gene [64,65]. As a result, we suggest using more than two different reference genes for standardization in future molecular experiments on *C. medinalis*. Moreover, some studies suggest that errors may be caused when more than three reference genes are used to normalize data [59]. Thus, the selection of reference genes for *C. medinalis* should be based on the appropriate number of reference genes in addition to stability.

## 5. Conclusions

In conclusion, the stability of thirteen candidate reference genes was analyzed by five reliable algorithms under different experimental conditions. The optimal combination of most stable reference genes was *PGK*, *RPL18,* and *EF1β* for developmental stages; *RPS3* and *RPL18* for larvae tissues; *EF1β* and *PGK* for larvae feeding on different rice varieties; *EF1α*, *EF1β*, and *PGK* for larvae temperature treatments; *PGK* and *RPL13* for adult ages; *PGK*, *EF1α*, and *ACT* for adult nutritional conditions; *RPL18* and *PGK* for adult mating statuses; *RPS3* and *PGK* for different adult take-off characteristics. *PGK* could be used as a reference gene of *C. medinalis* in most physiological conditions. Our results provide a basis for further studies on the expression of target genes in *C. medinalis* under these different experimental conditions. However, there was no single universal reference gene that could be used under all experimental conditions. The applicability of the reference genes recommended in this study under other experimental conditions remains to be determined.

## Figures and Tables

**Table 1:** Primer sequences and amplicon characteristics of the thirteen reference genes in *Cnaphalocrocis medinalis*.

Gene Name	Gene Symbol	Primer Sequence (5′ to 3′)	Product Size (bp)	Tm (°C)	Efficiency (%)	Regression Coefficient (R^2^)	Slope
Elongation factor 1 α	*EF1α*	F: CTGCTGTCGCTTTCGTCCC R: CTTGCCCTCAGCCTTACCCTC	122	55	105	0.992	−3.217
Arginine kinase	*AK*	F: CGCAACCCTCGAGAAATTGGA R: ACACCCGACTGGATGCAA	159	55	112	0.996	−3.071
Elongation factor 1 β	*EF1β*	F: CTTCTTACACTCCCGCCGAAC R: GCGTCCTCTTCCTCATCACC	154	55	108	0.996	−3.135
Glyceraldehyde-3-Phosphate dehydrogenase	*GAPDH*	F: CTGCCACTCAAAAGACCGT R: AAGGCCATACCAGTCAGT	154	53	104	0.992	−3.233
Phosphoglycerate kinase	*PGK*	F: CAGCCCTCATTGCAAAGTCC R: GCAGCTTGTTGATTCCATAACCA	162	57	109	0.999	−3.115
Ribosomal protein L 13	*RPL13*	F: ATCAACAGCCGTCAGATCG R: TTTCCATTGTGTGTCGCCTC	193	55	109	0.995	−3.117
Ribosomal protein L 18	*RPL18*	F: GGCGCACCGAAGTTAAATCTCA R: AGCCACGGTCATCTTAGGAAC	263	54	110	0.996	−3.111
Ribosomal protein S 3	*RPS3*	F: AGGTTCAACATCCCCGAGCA R: CGGACACAACAACCTCGCAAC	193	55	109	0.995	−3.114
18S ribosomal RNA	*18S rRNA*	F: TTTTATAATGCCGACGAAGCGAGA R: CCCGAAAGCCCTGAACCAC	155	56	104	0.990	−3.226
TATA-box binding protein 1	*TBP1*	F: AATGCTGAATACAACCCGAAG R: TCCTAGCAGCTAATCTTGAGT	142	55	108	0.982	−3.141
TATA-box binding protein 2	*TBP2*	F: ATAACCAATGCTGCAAACACC R: CGCTGTCTTTCATTTGTAGAACCA	128	55	108	0.996	−3.146
β-actin	*ACT*	F: CACACAGTGCCCATCTACGA R: GCGGTGGTGGTGAATGAGTA	125	55	102	0.998	−3.276
Ubiquinol-cytochrome c reductase	*UCCR*	F: ACAGTCGCCTTCAAAGCTGGT R: CCAATCTGTGCCAACTTGCGT	165	55	119	0.999	−2.937

**Table 2:** Ranking of the candidate reference genes in *C. medinalis* under different conditions.

Experimental Conditions	Ranking	ΔCt		BestKeeper		NormFinder		geNorm	
Developmental stages	1	*EF1β*	1.264	*GAPDH*	0.660	*RPL18*	0.537	*EF1* *β* *PGK*	0.632
	2	*PGK*	1.278	*EF1β*	0.798	*PGK*	0.570	*—*	—
	3	*RPL18*	1.286	*RPL18*	0.800	*EF1β*	0.587	*EF1α*	0.838
	4	*EF1α*	1.332	*EF1α*	0.840	*EF1α*	0.682	*RPL18*	0.745
	5	*ACT*	1.375	*PGK*	0.875	*ACT*	0.733	*ACT*	0.922
	6	*RPS3*	1.395	*RPS3*	0.911	*RPS3*	0.813	*RPS3*	0.955
	7	*GAPDH*	1.403	*RPL13*	0.997	*GAPDH*	0.858	*GAPDH*	0.996
	8	*TBP2*	1.470	*ACT*	1.005	*TBP2*	0.906	*TBP2*	1.053
	9	*TBP1*	1.594	*TBP2*	1.023	*TBP1*	1.127	*TBP1*	1.114
	10	*AK*	1.780	*TBP1*	1.302	*AK*	1.397	*RPL13*	1.204
	11	*RPL13*	1.813	*AK*	1.398	*RPL13*	1.469	*AK*	1.289
	12	*UCCR*	2.304	*UCCR*	1.426	*UCCR*	2.065	*UCCR*	1.440
	13	*18S rRNA*	2.449	*18S rRNA*	2.027	*18S rRNA*	2.339	*18S rRNA*	1.595
Larval tissues	1	*RPL18*	1.270	*EF1α*	0.624	*RPS3*	0.153	*18S rRNA* *TBP1*	0.371
	2	*RPS3*	1.283	*TBP2*	0.894	*RPL18*	0.153	*—*	—
	3	*TBP1*	1.318	*RPS3*	0.896	*EF1α*	0.219	*RPL18*	0.446
	4	*EF1α*	1.367	*EF1β*	0.990	*TBP1*	0.326	*RPS3*	0.490
	5	*18S rRNA*	1.414	*PGK*	1.006	*18S rRNA*	0.585	*PGK*	0.586
	6	*PGK*	1.457	*TBP1*	1.027	*PGK*	0.633	*EF1β*	0.640
	7	*EF1β*	1.508	*RPL18*	1.029	*EF1β*	0.738	*EF1α*	0.697
	8	*GAPDH*	1.557	*RPL13*	1.081	*GAPDH*	0.773	*RPL13*	0.761
	9	*RPL13*	1.626	*18S rRNA*	1.113	*RPL13*	0.933	*GAPDH*	0.832
	10	*AK*	1.802	*GAPDH*	1.125	*AK*	1.115	*AK*	0.941
	11	*TBP2*	1.837	*AK*	1.261	*TBP2*	1.121	*TBP2*	1.035
	12	*ACT*	4.052	*ACT*	3.144	*ACT*	3.944	*ACT*	1.494
	13	*UCCR*	4.054	*UCCR*	3.450	*UCCR*	3.949	*UCCR*	1.888
Larvae feeding on different rice varieties	1	*EF1β*	0.529	*PGK*	0.322	*EF1β*	0.177	*EF1β* *PGK*	0.247
	2	*PGK*	0.563	*EF1α*	0.328	*PGK*	0.249	*—*	—
	3	*EF1α*	0.597	*EF1β*	0.366	*18S rRNA*	0.293	*ACT*	0.284
	4	*TBP1*	0.597	*ACT*	0.420	*TBP1*	0.299	*EF1α*	0.296
	5	*18S rRNA*	0.604	*GAPDH*	0.435	*EF1α*	0.341	*RPL18*	0.324
	6	*ACT*	0.612	*RPL18*	0.458	*RPL18*	0.355	*TBP1*	0.371
	7	*RPL18*	0.619	*TBP1*	0.466	*ACT*	0.373	*18S rRNA*	0.399
	8	*GAPDH*	0.691	*18S rRNA*	0.513	*GAPDH*	0.441	*RPS3*	0.433
	9	*TBP2*	0.755	*RPS3*	0.532	*TBP2*	0.553	*GAPDH*	0.466
	10	*RPS3*	0.757	*TBP2*	0.533	*RPS3*	0.609	*TBP2*	0.509
	11	*RPL13*	0.868	*UCCR*	0.638	*RPL13*	0.699	*RPL13*	0.566
	12	*AK*	1.007	*AK*	0.735	*AK*	0.870	*AK*	0.635
	13	*UCCR*	1.209	*RPL13*	0.807	*UCCR*	1.114	*UCCR*	0.724
Larvae temperature treatments	1	*EF1β*	0.895	*EF1α*	0.387	*EF1β*	0.412	*EF1α* *PGK*	0.507
	2	*RPS3*	0.921	*PGK*	0.426	*TBP1*	0.464	*—*	—
	3	*EF1α*	0.924	*RPL18*	0.515	*TBP2*	0.489	*RPS3*	0.555
	4	*PGK*	0.933	*RPS3*	0.565	*EF1α*	0.506	*EF1β*	0.597
	5	*TBP1*	0.937	*EF1β*	0.648	*RPS3*	0.520	*TBP2*	0.659
	6	*TBP2*	0.942	*TBP1*	0.687	*PGK*	0.574	*TBP1*	0.684
	7	*GAPDH*	0.998	*GAPDH*	0.696	*GAPDH*	0.632	*GAPDH*	0.713
	8	*RPL18*	1.040	*RPL13*	0.740	*RPL13*	0.715	*RPL18*	0.735
	9	*RPL13*	1.056	*TBP2*	0.835	*RPL18*	0.743	*RPL13*	0.770
	10	*AK*	1.146	*ACT*	0.863	*AK*	0.885	*AK*	0.805
	11	*ACT*	1.211	*AK*	1.023	*ACT*	0.896	*ACT*	0.869
	12	*18S rRNA*	1.402	*18S rRNA*	1.291	*18S rRNA*	1.168	*18S rRNA*	0.953
	13	*UCCR*	1.925	*UCCR*	1.602	*UCCR*	1.803	*UCCR*	1.102
Adult ages	1	*PGK*	1.041	*UCCR*	0.807	*PGK*	0.202	*PGK* *RPL13*	0.404
	2	*RPL13*	1.110	*ACT*	0.892	*EF1α*	0.281	*—*	—
	3	*EF1α*	1.113	*GAPDH*	1.022	*RPL13*	0.302	*EF1α*	0.426
	4	*RPS3*	1.161	*EF1α*	1.158	*RPS3*	0.488	*EF1β*	0.505
	5	*EF1β*	1.197	*AK*	1.231	*EF1β*	0.538	*RPS3*	0.556
	6	*RPL18*	1.231	*RPL13*	1.374	*RPL18*	0.602	*RPL18*	0.615
	7	*TBP1*	1.392	*PGK*	1.396	*TBP1*	0.902	*TBP1*	0.739
	8	*UCCR*	1.546	*EF1β*	1.401	*UCCR*	1.136	*UCCR*	0.886
	9	*GAPDH*	1.668	*RPS3*	1.709	*GAPDH*	1.334	*ACT*	1.020
	10	*TBP2*	1.692	*RPL18*	1.744	*TBP2*	1.365	*GAPDH*	1.117
	11	*ACT*	1.712	*TBP1*	1.960	*ACT*	1.380	*TBP2*	1.203
	12	*AK*	2.134	*TBP2*	2.075	*AK*	1.960	*AK*	1.335
	13	*18S rRNA*	2.314	*18S rRNA*	2.919	*18S rRNA*	2.184	*18S rRNA*	1.485
Adult nutritional conditions	1	*PGK*	1.024	*ACT*	0.379	*PGK*	0.169	*EF1α* *PGK*	0.338
	2	*EF1α*	1.061	*EF1β*	0.520	*EF1α*	0.310	*—*	—
	3	*RPL13*	1.140	*EF1α*	0.594	*RPL13*	0.380	*RPL18*	0.481
	4	*RPL18*	1.152	*RPL18*	0.710	*RPL18*	0.512	*ACT*	0.554
	5	*RPS3*	1.205	*AK*	0.718	*RPS3*	0.512	*RPL13*	0.628
	6	*ACT*	1.214	*PGK*	0.808	*GAPDH*	0.570	*GAPDH*	0.709
	7	*GAPDH*	1.221	*UCCR*	0.896	*TBP1*	0.627	*RPS3*	0.768
	8	*TBP1*	1.256	*GAPDH*	0.911	*ACT*	0.721	*TBP1*	0.807
	9	*EF1β*	1.351	*RPL13*	1.000	*EF1β*	0.952	*EF1β*	0.857
	10	*AK*	1.411	*TBP1*	1.377	*AK*	1.013	*AK*	0.904
	11	*TBP2*	1.609	*RPS3*	1.430	*TBP2*	1.232	*TBP2*	1.004
	12	*UCCR*	2.236	*TBP2*	1.957	*UCCR*	2.145	*UCCR*	1.173
	13	*18S rRNA*	2.982	*18S rRNA*	3.395	*18S rRNA*	2.933	*18S rRNA*	1.451
Adult mating statuses	1	*RPL18*	1.230	*UCCR*	0.560	*RPL18*	0.088	*EF1α* *PGK*	0.210
	2	*PGK*	1.248	*AK*	0.707	*RPL13*	0.249	*—*	—
	3	*ACT*	1.271	*RPS3*	1.105	*PGK*	0.268	*EF1β*	0.261
	4	*RPL13*	1.286	*ACT*	1.260	*ACT*	0.412	*RPL18*	0.277
	5	*EF1β*	1.295	*EF1β*	1.465	*EF1β*	0.478	*ACT*	0.331
	6	*EF1α*	1.316	*RPL18*	1.488	*EF1α*	0.518	*RPS3*	0.398
	7	*RPS3*	1.341	*EF1α*	1.555	*RPS3*	0.576	*RPL13*	0.454
	8	*GAPDH*	1.558	*PGK*	1.573	*GAPDH*	0.668	*GAPDH*	0.608
	9	*TBP2*	2.027	*GAPDH*	1.583	*TBP2*	1.540	*TBP2*	0.887
	10	*TBP1*	2.102	*RPL13*	1.717	*TBP1*	1.660	*TBP1*	1.065
	11	*AK*	2.234	*TBP2*	3.040	*AK*	1.974	*AK*	1.224
	12	*UCCR*	2.669	*TBP1*	3.116	*UCCR*	2.564	*UCCR*	1.433
	13	*18S rRNA*	3.814	*18S rRNA*	4.845	*18S rRNA*	3.770	*18S rRNA*	1.799
Different adult take-off characteristics	1	*RPS3*	0.374	*ACT*	0.208	*RPS3*	0.085	*PGK * *RPS3*	0.093
	2	*PGK*	0.391	*UCCR*	0.240	*PGK*	0.186	*—*	—
	3	*RPL18*	0.410	*EF1α*	0.258	*RPL13*	0.203	*ACT*	0.156
	4	*EF1α*	0.414	*AK*	0.276	*RPL18*	0.222	*EF1α*	0.165
	5	*ACT*	0.419	*PGK*	0.310	*EF1α*	0.237	*RPL18*	0.188
	6	*RPL13*	0.423	*RPS3*	0.340	*ACT*	0.264	*RPL13*	0.212
	7	*TBP1*	0.463	*RPL18*	0.364	*TBP1*	0.278	*UCCR*	0.247
	8	*TBP2*	0.478	*RPL13*	0.392	*TBP2*	0.312	*AK*	0.277
	9	*18S rRNA*	0.524	*TBP2*	0.581	*18S rRNA*	0.381	*TBP1*	0.316
	10	*UCCR*	0.535	*TBP1*	0.592	*UCCR*	0.459	*TBP2*	0.347
	11	*AK*	0.569	*18S rRNA*	0.661	*AK*	0.483	*18S rRNA*	0.375
	12	*EF1β*	0.672	*EF1β*	0.668	*EF1β*	0.566	*EF1β*	0.424
	13	*GAPDH*	1.007	*GAPDH*	0.973	*GAPDH*	0.957	*GAPDH*	0.514

## Data Availability

The data presented in this study are available in the article.

## Supplementary Materials

The following supporting information can be downloaded at: https://www.mdpi.com/article/10.3390/insects13111046/s1, Figure S1: Amplification specificity of primers in PCR. Figure S2: Melting curves from RT-qPCR of the candidate genes.
