# Supplementary material for: Reference Genes for Expression Analysis Using RT-qPCR in Cnaphalocrocis medinalis (Lepidoptera: Pyralidae)
Source: Insects. 2022 Nov 13;13(11):1046. doi: 10.3390/insects13111046 (PMC9697642; doi:10.3390/insects13111046)
Supplement: Supplementary file 1 [file insects-13-01046-s001.zip › Supplementary Figures.pdf]

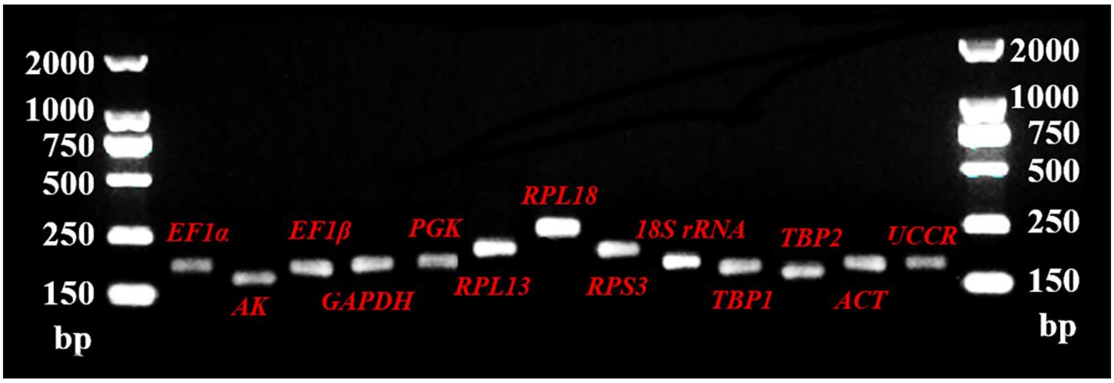

Supplementary Figure S1. Amplification specificity of primers in PCR.

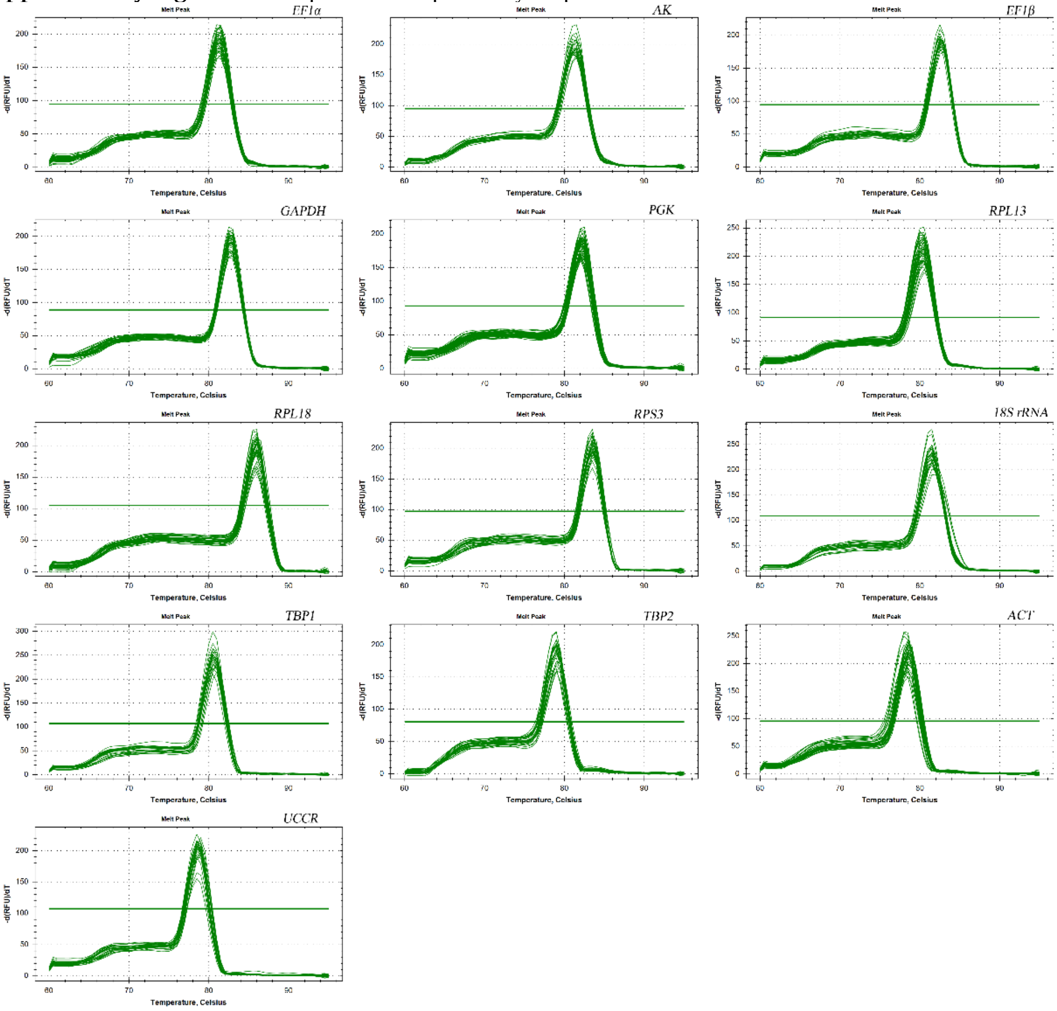

Supplementary Figure S2. Melting curves from RT-qPCR of the thirteen candidate genes.
